# Functional Annotation of a Full-Length Transcriptome and Identification of Genes Associated with Flower Development in *Rhododendron simsii* (Ericaceae)

**DOI:** 10.3390/plants10040649

**Published:** 2021-03-29

**Authors:** Qunlu Liu, Fiza Liaquat, Yefeng He, Muhammad Farooq Hussain Munis, Chunying Zhang

**Affiliations:** 1 Department of Landscape Architecture, School of Design, Shanghai Jiao Tong University, Shanghai 200240, China; liuql@sjtu.edu.cn (Q.L.); yf@sjtu.edu.cn (Y.H.); 2 School of Agriculture and Biology, Shanghai Jiao Tong University, Shanghai 200240, China; fiza.liaquat@bs.qau.edu.pk; 3 Department of Plant Sciences, Faculty of Biological Sciences, Quaid-i-Azam University, Islamabad 45320, Pakistan; munis@qau.edu.pk; 4 Shanghai Engineering Research Center of Sustainable Plant Innovation, Shanghai Botanical Garden, Shanghai 200231, China

**Keywords:** *Rhododendron simsii*, transcriptome, full length transcript, alternative splicing

## Abstract

*Rhododendronsimsii* is one of the top ten famous flowers in China. Due to its historical value and high aesthetic, it is widely popular among Chinese people. Various colors are important breeding objectives in *Rhododendron L*. The understanding of the molecular mechanism of flower color formation can provide a theoretical basis for the improvement of flower color in *Rhododendron L*. To generate the *R.* *simsii* transcriptome, PacBio sequencing technology has been used. A total of 833,137 full-length non-chimeric reads were obtained and 726,846 high-quality full-length transcripts were found. Moreover, 40,556 total open reading frames were obtained; of which 36,018 were complete. In gene annotation analyses, 39,411, 18,565, 16,102 and 17,450 transcriptions were allocated to GO, Nr, KEGG and COG databases, correspondingly. To identify long non-coding RNAs (lncRNAs), we utilized four computational methods associated with Protein families (Pfam), Cooperative Data Classification (CPC), Coding Assessing Potential Tool (CPAT) and Coding Non Coding Index (CNCI) databases and observed 6170, 2265, 4084 and 1240 lncRNAs, respectively. Based on the results, most genes were enriched in the flavonoid biosynthetic pathway. The eight key genes on the anthocyanin biosynthetic pathway were further selected and analyzed by qRT-PCR. The F3′H and ANS showed an upward trend in the developmental stages of *R. simsii*. The highest expression of F3′5′H and FLS in the petal color formation of *R. simsii* was observed. This research provided a huge number of full-length transcripts, which will help to proceed genetic analyses of *R.* *simsii*. native, which is a semi-deciduous shrub.

## 1. Introduction

*Rhododendron* is one of the largest genera in the Ericaceae family, with several species having high horticultural value and being grown as ornamental plants all over the world. The genus contains 580 species in six subgenera in China, with approximately 420 being endemic, including several species described after the publication of the Flora of China [1]. Numerous varieties of *Rhododendron* have been cultured, and it has become one of the most popular flowering shrubs in the world. Their colorful flowers are the most attractive traits. Plant flower color is the result of the synergistic effect of many factors, but it is fundamentally due to the presence of specific pigments in petal cells [2]. At present, the plant pigments can be divided into four categories: flavonoids, carotenoids, chlorophyll, and alkaloid pigments. The flower color is mainly affected by the first three kinds of flavonoids. Flavonoids are the main pigments, most of which exist in plant vacuoles, so that the petals show red, purple red and other colors [3]. Flavonoids (flavonoids) are the most widely existing plant polyphenols in nature, which are classified according to their structural characteristics, including anthocyanin (anthocyanin), flavonoids alcohol (flavonols), flavonoids (flavones), etc. [4]. Thin-layer chromatography was previously used to explore the flavonoid pigments that contribute to petal coloration in azalea cultivars and contain the anthocyanins cyanidin 3-O-galactoside, cyanidin 3-O-glucoside, cyanidin 3-O-arabinoside, cyanidin 3,5-di-O-glucoside, cyanidin 3-O-arabinoside-5-O-glucoside, peonidin 3,5-di-O-glucoside and malvidin 3,5-di-O-glucoside, as well as flavonols azaleatin, quercetin, myricetin and their glycoside derivatives [5].

Anthocyanins are the main pigment groups that determine the color of plant flowers, the most common of which include (cyanidin), (pelargonidin), (petunidin), (delphindin), (peonindin), etc. [6]. It was considered that the anthocyanins contained (Cy), (Dp), (Pe), (Mv) and (Pt), which were usually in the form of anthocyanins. Anthocyanin is the most concerned natural plant pigment in the related research of flower color improvement, and its biosynthesis pathway is also one of the clearest secondary metabolic pathways in plants [7]. Recent discoveries have enabled genome and transcriptome knowledge in numerous species. However, *R. simiisi* genome and transcriptome sequencing has lagged behind that of other organisms, and knowledge of the sequence and structure of its genes is poor. As a result, the generation of transcriptome data may provide a significant molecular biology foundation for the reanalysis.

However, up to date, no researcher has studied the transcriptomic and full-length transcript of *R. simissi* using single-molecule long-read sequencing. Recent discoveries demonstrate that PacBio’s single-molecule real-time (SMRT) sequencing technology is a more efficient method of obtaining full-length transcripts [8]. Both model and non-model organisms have had their transcriptomes analyzed using SMRT sequencing technology [9]. Furthermore, transcriptome sequencing is a low-cost and easy strategy for developing large-scale SSRs. Many non-model plants have confirmed the discovery of SSR markers through RNA sequencing and their effective use in genetic improvement in recent years [10,11,12]. Since the establishment of large scale sequencing technology, to study gene expression regulation transcripts sequencing has become a key source, after the whole genome sequencing of humans was completed [13]. With its advantage of long-read length, high-quality full-length transcript information can be directly obtained to identify simple sequence repeat (SSR) and fulfill the functional annotation of transcripts and long non-coding RNAs prediction (lncRNA) [14,15].

In this study, Single Molecule Real-Time (SMRT) sequencing was performed to generate a full-length transcriptome of *R. simissi*. Based on the acquired transcriptome data, we performed functional annotations of transcripts, transcript factors (TFs) and simple sequence repeat (SSR) analysis, and lncRNA prediction.

Through the analysis of the expression pattern of differentially expressed genes, this provides important significance for further analyzing the molecular mechanism of the color formation of *Rhododendron* and promoting the cultivation and breeding of *Rhododendron* in China. This research may be a useful resource for further investigation of molecular mechanisms related to *R. simissi*.

## 2. Materials and Methods

### 2.1. Plant Materials

**Figure 1:**
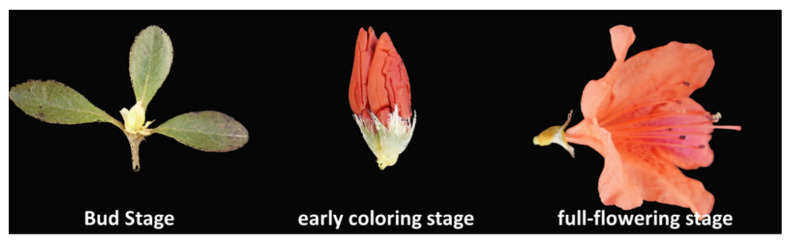
Three developmental stages of *R. simsii* flower.

Line “Y4” of *Rhododendron simsii* with cardinal flowers (Red-group 39B) was utilized as materials in this article. The 10-year-old “Y4” plants were cultivated in Shanghai botanical garden (31°08′46.88″ N, 121°26′39.85″ E), Shanghai, China. According to [16], the development of the *R. simsii* flower was divided into bud, early coloring and full-flowering stages (Figure 1). The fresh petals at the three stages were immediately preserved in liquid nitrogen after collection, and stored at −80 °C until extraction of RNA.

### 2.2. RNA Extraction

Plant tissues (0.2 g petals) were used for the extraction of RNA by using RNeasy plus Mini Kit (Qiagen, Valencia, CA, USA). After observing RNA on agarose gel, its quality and quantity were determined using Qubit 2.0 (Thermo Fisher Scientific, Waltham, MA, USA). RNA quality was also distinctly described by Qubit^®^ RNA Assay and RNA 6000 Nano Assay Kit. For PacBio sequencing, all the samples were present in ≥300 ng/µL concentration and >1µg RNA was used for sequencing.

### 2.3. Library Construction and SMRT Sequencing

For sequencing, C2 sequencing reagents were used in Pacific Biosciences (PacBio) real-time sequencer. Purified RNA was used to synthesize cDNA using SMRT PCR Synthesis Kit (Clontech, San Jose, CA, USA). Full-length cDNA of different sizes was selected and cDNA libraries were constructed using the BluePippin^®^ (SageScience, Beverly, MA, USA). After BluePippin screening, the fragments were subjected to large-scale PCR to obtain sufficient total cDNA and quantified by using Qubit fluorometer (Life Technologies, Carlsbad, CA, USA). The libraries’ uniqueness was maintained by using the Agilent Bioanalyzer 2100 system, and SMRT sequencing was achieved.

### 2.4. Error Correction and Quality Filtering

Sequence statistics were obtained by using the SMRTlink 5.1 software. To create CCS by subread BAM files, specific parameters such as min_length 50, min_zscore −9999.0, min_passes 2, max_drop_fraction 0.8, no_polish TRUE, min_predicted_accuracy 0.8, and max_length 15,000 were followed. The output was CCS.BAM filest, which were categorized into full length and non-full length reads by using pbclassify.py. Non-full length and full-length FASTA files produced were then fed into cluster step, and extra nucleotide errors in the consensus reads were corrected by LoRDEC software. Any redundancy in corrected consensus reads was removed by CD-HIT (-c 0.95 -T 6 -G 0 -aL 0.00 -aS 0.99) to obtain final transcripts for the subsequent analysis.

### 2.5. Functional Annotation of Transcripts

We recognized functional annotations matching each unique transcript by searching NR [17], NT, Protein families (Pfam) (http://pfam.xfam.org) KOG (http://www.ncbi.nlm.nih.gov/COG/) [18], Swiss-prot (http://www.ebi.ac.uk/uniprot/) [19], KEGG (http://www.genome.jp/kegg/) [20] and GO [21]. We used BLAST software (with “1e-10” e-value) in the NT database using Diamond BLASTX v2.7.1 (ftp://ftp.ncbi.nlm.nih.gov/blast/executables/blast+/LATEST/). The same e-value (“1e-10”) was set in NR, KOG, Swiss-Prot, KEGG and Pfam database analysis.

### 2.6. Identification of TFs, lncRNAs and SSR

Plant transcription factors were predicted using iTAK v1.7a (https://github.com/kentnf/iTAK/) [22]. Four tools, CNCIv2 (https://github.com/www-bioinfo-org/CNCI) [23], CPCvcpc-0.9-r2 (http://cpc.cbi.pku.edu.cn/) with e-value “1e-10” [24], Pfam-scan (E 0.001 -domE 0.001) [25] and PLEKv1.2 (https://sourceforge.net/projects/plek/) with min length 200 [26], were chosen to predict candidate long non-coding RNAs (lncRNAs). Transcripts predicted with coding potential by either/all of the above mentioned tools were filtered out, and those without coding potential were considered as a candidate set of lncRNAs. Transcript sequences were examined for homology via searches against the non-redundant nucleotide database (Nr) 25, Swiss-Prot protein26, protein family (pfam) 27, non-supervised orthologous groups (eggNOG)28, clusters of orthologous groups of proteins (COG)29, eukaryotic ortholog groups (KOG) 30, gene ontology (GO) 31, kyoto encyclopedia of genes and genomes (KEGG) 32 databases with BLAST alignment (E-value ≤ ^10−5^).

### 2.7. Development of SSR Markers

SSRs were identified by MISA v1.0 (http://pgrc.ipk-gatersleben.de/misa/) [27], with default parameters. MISA can recognize seven SSR types (mono, di, tri, tetra, penta and hexa nucleotide compound SSR) by analyzing transcript sequences.

### 2.8. qRT-PCR Analysis

The fresh petals at the three stages were used for RNA extraction via RNeasy plus Mini Kit (Qiagen, Valencia, CA, USA). The cDNA was synthesized by using SMART PCR cDNA synthesis kit (Clontech, CA, USA). In the reaction mixture of qPCR, SYBR green (10 µL), ddH2O (7.2 µL), primers (0.4 µL) and cDNA (2.0 µL) were used. The qPCR reaction was performed at 94 °C for 40 s followed by 30 cycles of 94 °C for 10s, 94 °C for 40 s, 54 °C for 30 s and 72 °C for 90 s. At the end, the reaction was kept at 72 °C for 5 min. The primers used for PCR are listed in Table 1.

## 3. Results and Analysis

### 3.1. Single Molecule Real Time Sequencing (SMRT)

The qualified RNAs extracted from petals of *R. simsii* “Y” flowers at bud, early coloring and full-flowering stages were used to construct the full-length cDNA library (1–2 kb, 2–3 kb, and >3 kb). Single molecular real time (SMRT) sequencing was performed to generate the continuous long reads exploiting Pacbio RS II platform. Total 40G clean data were obtained from the qualified polymerase reads (length > 50 bp, accuracy > 0.90) after the adapter sequences were removed. According to the full passes number (≥ 1) and accuracy (> 0.90), 833,137 circular consensus (CCS) reads were screened out, and the average length of CCS read was 2758 bp (Table 2). Among the total CCS reads, 87.24% (726,846) of reads were identified as the full length non-chimeric (FLNC) reads. Then, the FLNC reads were analyzed with the algorithm of iterative clustering to obtain 71,727 consensus isoforms. After error correction and polishing, 71,210 high-quality (> 0.99) consensus isoforms were filtrated. The low-quality consensus isoforms were proofread with the clean data obtained by Illumina next generation sequencing. After reducing the redundancy, 41,112 full-length unigenes were filtrated from the consensus isoforms.

### 3.2. Open Reading Frame and Alternative Splicing Event Prediction

**Figure 2:**
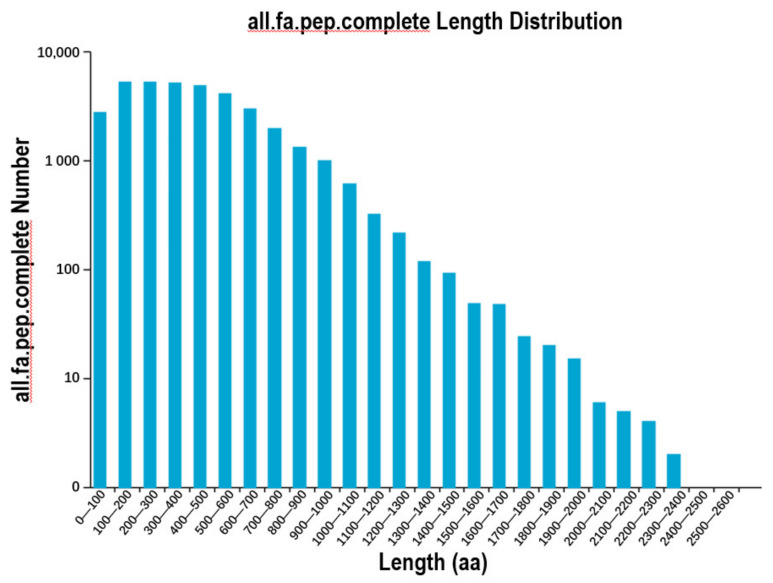
Distribution of predicted CDS encoded protein length.

Using the software TransDecoder, 40,566 Open Reading Frames (ORF) were to be found. The sum of 36,018 complete ORFs and the length distribution of the complete ORFs were analyzed (Figure 2). Around all transcripts acquired by SMRT sequencing, 2844 alternative splicing (AS) events were examined (Table S1). Due to the absence of an available *R. simsii* reference genome, further characterization of the types of AS events would be warranted in predicted studies.

### 3.3. Long Non-Coding RNA Identification

**Figure 3:**
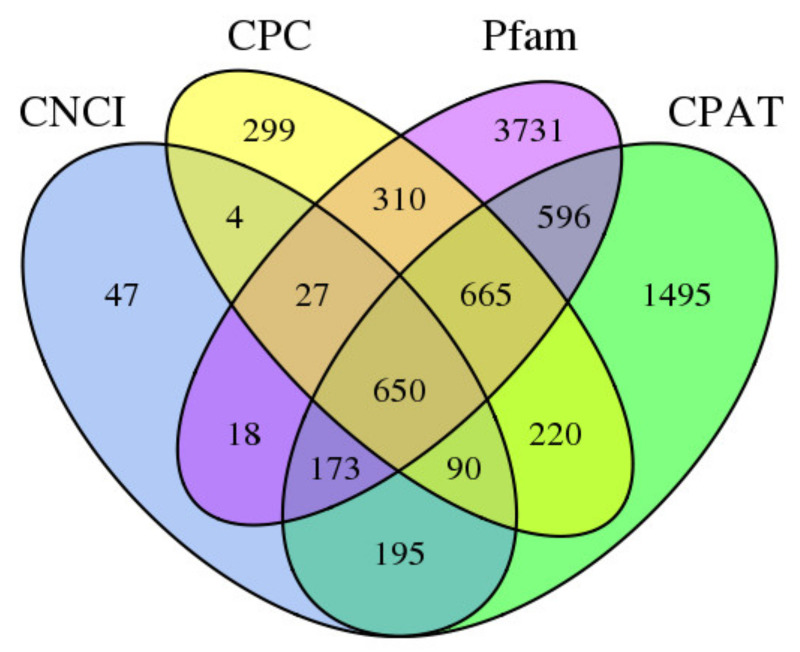
Venn diagram of long non-coding RNAs (lncRNAs) CPC: coding potential calculator; CNCI: coding-noncoding index; CPAT: coding potential assessment tool; Pfam: s protein families.

Long non-coding RNAs (lncRNAs) are a class of poly-A non-coding RNAs that play roles in three stages of the plant. In this research, four computational approaches were used to recognize lncRNAs, including Coding Assessing Potential Tool (CPAT), Cooperative Data Classification (CPC), Coding Non Coding Index (CNCI) and Pfam databases. A total of 1104, 2265, 6170 and 4084 lncRNAs were recognized in the CPAT, CNCI, CPC, and Pfam databases, respectively (Table S2). By screening transcripts of less than 300 bp, 5155 transcripts were evaluated as lncRNAs by all methods (Figure 3).

### 3.4. Transcription Factor Prediction

**Figure 4:**
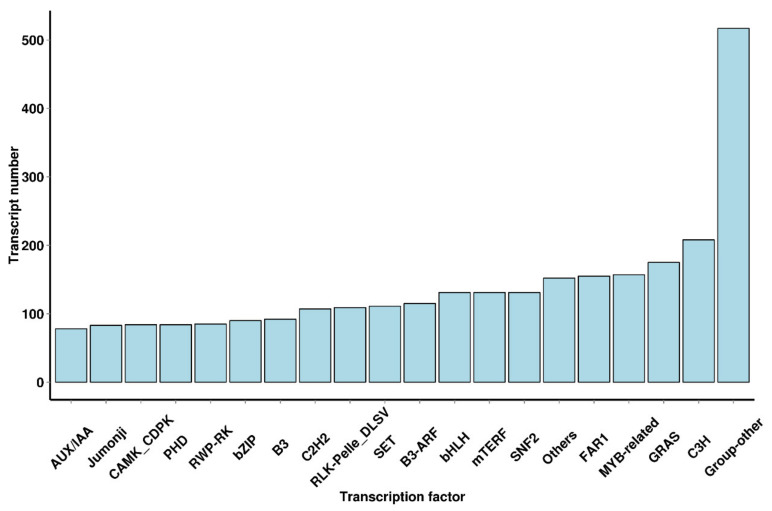
Distribution of transcription factor types.

TFs play a major role in plant growth development and are the key regulators of gene expression. According to the recent work, 6005 putative TFs were allocated and classified into 64 families (Table S3). The TFs in the *R. simsii* transcriptome mainly belong to the CH3 (208, 3.46%), GRAS (175, 2.91%), MYB-related (222, 3.70%), FAR1 (155, 2.58%), SNF2 (131, 2.18%), mTERF (131, 2.18%), bHLH (131, 2.18%), B3-ARF (115, 1.92%), SET (111, 1.85%), and RLK-Pelle-DLSV (109, 1.78%) families (Figure 4).

### 3.5. Functional Annotation of Transcripts

All 726,846 unique SMRT transcripts were functionally annotated by seven data storages, such as gene ontology (GO), eukaryotic ortholog groups (KOG), Protein family (Pfam), Swissport (15), COG (17), COG (18) and KEGG (20) by using BLAST (7) software (version 2.2.26) (Table 3).

**Figure 5:**
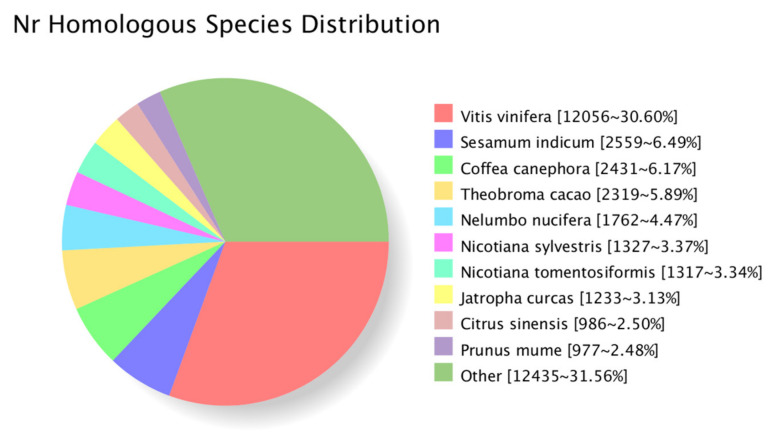
The classification statistics of Nr-annotated species in transcripts.

By comparing the transcript sequence to NR with homologous species, among them are *Vitis vinifera* (12,056), *Sesamum indicum* (2559), *Coffea canephora* (2431) and *Theobroma cacao* (2319), which were the top four distributed species of transcripts, as shown in Figure 5.

Function annotation of the non-redundant unigenes was conducted by searching against the main databases. A total of 95.86% (39,411) of the non-redundant unigenes were annotated in NR, 18,565 unigenes in GO, 16,102 unigenes in COG, 38,933 unigenes in eggNOG, 17,450 unigenes in KEGG, 25,787 unigenes in KOG, 34,156 unigenes in Pfam, and 29,498 unigenes in Swissport. In total, 96.13% (39,521) non-redundant unigenes were annotated, and more than 90% of the unigenes were longer than 1Kb (Table 4).

### 3.6. GO Classified Transcripts

**Figure 6:**
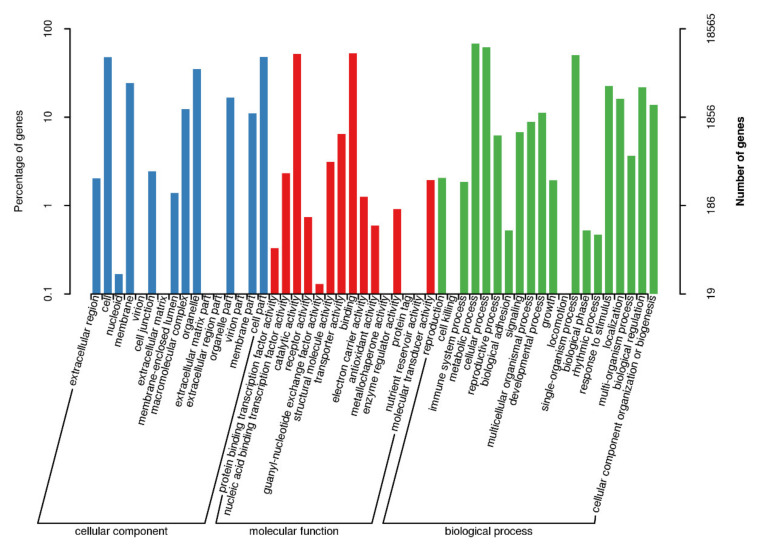
Transcript GO annotation classification statistics graph.

Transcripts’ GO classification statistics demonstrated 18,565 unique genes, which were enriched in major categories of molecular function, cellular component, biological component and catalytic activity (Figure 6). This analysis also helped us to obtain transcripts’ COG classification statistics.

### 3.7. COG Function Classification

**Figure 7:**
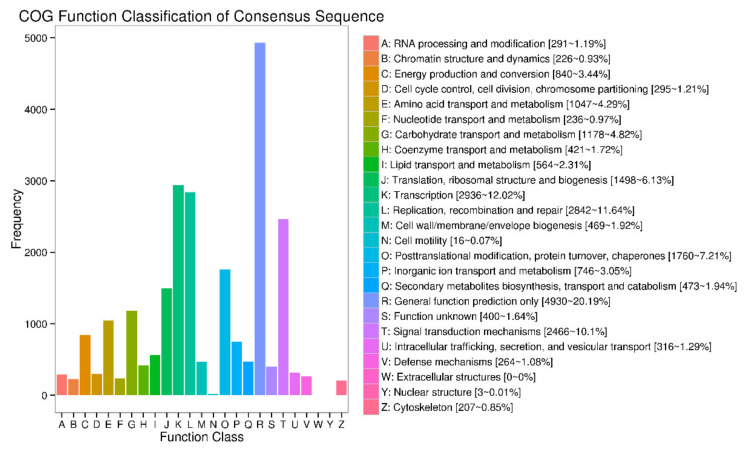
COG annotation classification statistics of transcripts.

To further research the functional classification of *R. simsii*, all transcripts were subjected to a search against the Clusters of COG database. This analysis indicated that 16,102 transcripts were allocated to 24 groups (Figure 7). The highest category was general function prediction (4930, 20.19%), followed by transcription (2936, 12.02%), and afterwards replication recombination and repair (2842, 11.64%). The six groups were in the range of less than 1%, including extracellular structure, nuclear structure and cell motility, nucleotide transport and metabolism and chromatin structure and dynamics.

### 3.8. KEGG Annotated Transcripts

KEGG data storage interpreted a total of 67,426 sequences and plotted 367 operative categories in *R. simsii*. Among them, metabolism was the largest category. The functional annotations of all 78,559 unique transcripts were detected in this study (Table S4). A large number of genes, especially interrelated in the salt-tolerance and fatty acid component of *S. superba*, were annotated, such as oxidative phosphorylation (1073), plant hormone signal transduction (506), fatty acid biosynthesis (246), the biosynthesis of unsaturated fatty acids (94) and α-linolenic acid metabolism (199). We also identified matches to our unique transcripts in clusters of orthologous groups of proteins (COG) (44,376, 56.49%), Pfam database (41,535, 52.87%) and Swiss-Port (58,535, 74.51%) (Table 5).

### 3.9. Expression Analysis in qRT PCR

**Figure 8:**
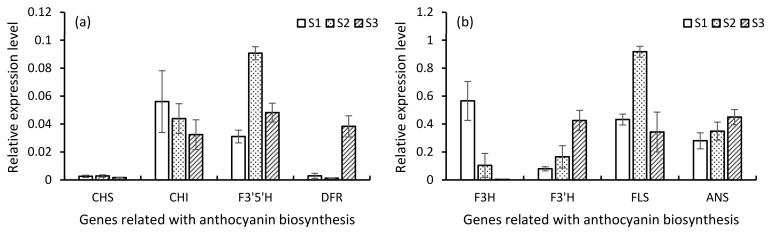
(**a**,**b**) Comparison of expression profiles of eight representative genes measured by qRT-PCR.

For the expression analysis of potential high expression genes, the anthocyanin pathway was selected for qRT analysis. A total of 67 transcripts were recognized through KEGG analysis (Table S5). Among them, nine genes related to anthocyanin were selected for qRT PCR analysis (Table S6). The results of qRT PCR analysis showed that the expression level of CHS in the bud stage increased continuously and reached the highest level in the early flowering stage, and then decreased in the flowering stage (Figure 8a). CHI shows higher expression in the bud stage than CHS (Figure 8a). The expression of F3H in the flowering period was highest, and then decreased (Figure 8b). These upstream genes have low expression in the blooming period, so we can speculate that the expression of upstream genes in promoting the biosynthesis of anthocyanin before the blooming period was completed. The downstream genes ANS and DFR are the genes directly regulating the synthesis of anthocyanin, which maintain a high expression level in the flower development stage (Figure 8).

## 4. Discussion

Since the development of high-throughput sequencing technology, transcriptomic analysis has become a valuable technology to study gene expression and regulation. However, due to the read length limitation of the second-generation sequencing in different organisms, the full-length transcript obtained by splicing is not complete. SMRT sequencing technology has effectively solved this problem.

In the past few years, the sequencing technology of three generations of full-length transcripts, represented by the PacBio platform, has slowly entered the field of vision. Compared with RNA-Seq sequencing technology, this sequencing technology avoids PCR amplification and reduces the cost and sequencing time, realizes the reaction speed and continuity of DNA polymerase in itself, and can directly measure RNA sequences with high accuracy [28]. As most non-model organisms lack genome data, it is particularly important to obtain full-length transcriptional group sequencing data. Full-length transcripts can greatly promote the basic and applied research on gene function, gene expression regulation and evolutionary relationships in these species [29]. With the maturity of the third generation sequencing technology and the great decrease in cost, full-length transcriptional group sequencing technology has been gradually applied in some plant transcriptional group research cases in recent years [30].

In past years, the PacBio platform sequenced the full-length transcriptional group of tea (Camellia sinensis) [31]. Finally, 213,389 polished consensus sequences were obtained, 223,120 CDS sequences were predicted, 195,062 SSR loci were detected, and 5785 transcription factors belonging to 60 transcription factor families were predicted.

The PacBio RS II platform to analyze the full-length transcription group of sunflowers, the 10.43Gb clean data, was obtained, and 38,302 de-redundant sequences were obtained [32]. The 44 differentially expressed genes were divided into eight families, all of which were involved in the biosynthesis of flavonoids. This study is the first transcriptome analysis using SMRT sequencing technology in *R. simiisi*. The sequencing peaks were gained from samples of leaves using the Pacific Biosciences Iso-Seq platform. A mixed pool of an equal amount of RNA from three developmental stages in *R. simsii* generated 40 Gb clean data from the PacBio platform. These data were corrected by Illumina clean reads after the integration and quality control of data from the two platforms. A total of 39,510 unigenes were annotated, and 6005 transcription factors were identified.

After sequencing, 833,137 circular consensus (CCS) reads were obtained, including 726,846 full-length reads non-chimeric (FLNC) sequences. The full-length non-chimeric sequences were clustered to obtain 71,727 consensus sequences, and 71,210 high-quality consensus sequences were obtained by polishing the consensus sequences. The low-quality consensus sequences were corrected with the second-generation transcriptome data, and they were consistent with high-quality. The sequences were merged and subjected to de-redundancy analysis to obtain 41,112 transcript sequences. Each sample obtained 2844 alternative splicing events, and a total of 44,205 SSR and 36,018 complete CDS regions were obtained.

Among some of the foremost important ornamental characteristics of *Rhododendron* is flower color. Recent studies indicated that (CHS, CHI, F3H, DFR, ANS, FLS, F3’H, F3’5’H) are the key enzymes involved in the anthocyanin biosynthesis pathway [33]. For this research, a sum of 67 key enzymes were allocated related to anthocyanin, and to analyze the expression eight key genes (*RhCHS*, *RhCHI*, *RhF3H*, *RhDFR*, *RhANS*, *RhFLS*, *RhF3’H*, *RhF3’5’H*) involved in the anthocyanin biosynthesis pathway, they were selected for subsequent analysis. Based on the data of sequencing, eight key enzyme genes were analyzed. It can be seen that the genes showing obvious differences mainly lie in the process from bud to early flowering stage, which is consistent with the significant change in expression at the early flowering stage, and most of the differentially expressed genes have shown quite high expression at the bud stage. It can be inferred that the expression of the flower color synthesis gene is before the formation of flower buds. In order to further understand the difference in the expression patterns of the screened structural genes and transcription factors in the process of color formation, the co-expression patterns of the screened structural genes were analyzed. The results showed that the expression of CHS increased continuously at the bud stage, reached the highest level at the beginning of flowering, and then decreased sharply at the flowering stage. According to the results [34] in *R. pulchrum* at S1 stage, five genes (CHS, CHI, F3H, ANS and DFR) were expressed, and the expression of CHS, F3H and ANS was the highest at the S1 stage and decreased during the development of the flower. The expression trend of CHI at bud stage was the same as that of CHS. CHI already showed ultra-high expression at the bud stage, and then decreased significantly at the flowering stage. The same as other results [35], our results also shown that the expression of F3H was the highest at the bud stage and then decreased, and these upstream genes decreased to the lowest level at the flowering stage. Downstream genes ANS and DFR are genes that directly regulate anthocyanin synthesis, maintain high expression at the flower development stage, and show an upward trend from the bud stage to the flowering stage, but do not change obviously from the early flowering stage to the flowering stage, indicating that the coloring stage was completed before the early flowering stage. The expression levels of DFR, FLS and ANS were markedly higher at early stages than at later stages, according to an analysis of anthocyanin biosynthesis-related genes [36]. As a key gene involved in the synthesis of flavonoids, the expression of the FLS gene reached the highest level from the bud stage to the early flowering stage and then decreased during the development of petals, indicating that the biosynthesis of flavonoids was completed at the early flowering stage, and flavonoids affected the formation of flower color. This study has provided enriched information about the expression and regulation of genes from the bud to flowering stage of *R. simiisi*. In addition, this study provided for the first time a full length transcriptome of *R. simsii* using the SMRT sequencing method.

## 5. Conclusions

In this study, we first used PacBio platform based third-generation sequencing technology combined with full-length transcriptome sequencing of a mixed petal sample from three developmental stages of *Rhododendron simiisi*. A total of 41,112 sequences were obtained. The full-length transcripts were compared with major databases, and 39,510 sequences were annotated. KEGG has obtained a relatively complete biosynthetic pathway of anthocyanins of *Rhododendron*, which provides the basis for follow-up studies of anthocyanin metabolism in *Rhododendron*. By functional annotation analysis of full-length transcripts, 67 key enzyme genes were screened out. These genes may be related to the formation of *R. simiisi*. These structural genes and regulatory genes are the important research objects of our next differential gene expression analysis. The transcriptome design in this research will assist future research on functional genomics and facilitate support for advanced genetic engineering of *R. simiisi*.

## Figures and Tables

**Table 1:** The primers used for qRT-PCR analysis.

Primer ID	Gene ID	Forward Primer	Reverse Primer
*RhCHS*	F01_transcript/61502	GCTTACCGTCGAGACCGTGG	AACAATGGGCCTCACCAGCC
*RhCHI*	F01_transcript/68926	GCCGCGTTGGAGCAAATTGT	CCCCCTGGTTCCACCCAAAT
*RhF3H*	F01_transcript/64242	CTCACTCTCGGCCTCAAGCG	TGCCTCAGGGGCTGGATTCT
*RhDFR*	F01_transcript/64345	GGCTTCATCGGCTCATGGCT	TCCGCTTTGGGCAACTCCAG
*RhANS*	F01_transcript/40426	CTCCTTCTCCTCCACCGGCT	GGGGGTTGTTCCAGGTGGTG
*RhF3’H*	F01_transcript/54870	CGATCCCCACCACTCCATC	AAGAACTGTGCGGCAACCGA
*RhF3’5’H*	F01_transcript/50526	TGCTGACTACGGCCCGAGAT	GGTATCACCACTGCCTCGCC
*RhMYB1*	F01_transcript/63169	ACTCGAGCTGTAGCCCACCA	CGTTCGGAAGACGAGCCTCC
*RhMYB2*	F01_transcript/66405	GGCAACTGGAGGTCTCTGCC	CCGGCAGCCTTCCTGCTATG
*RhbHLH1*	F01_transcript/65059	CCCTTTCGGCTATGCTGCGA	CCCTTTCGGCTATGCTGCGA
*RhbHLH2*	F01_transcript/50357	CTTGGTTGCTTCGGCCTCCA	ACCTCACTCCCTCTCGCCTC

**Table 2:** Summary of reads of inserts from single-molecule long-read sequencing.

Samples	cDNA Size	CCS Number	Read Bases of CCS	Mean Read Length of CCS	Mean Number of Passes
F01	1–6 k	833,137	2,298,485,998	2758	19

**Table 3:** Summary of functional annotation of *S. superba* transcriptome.

Annotated Databases	Isoform Number
COG	16,102
GO	18,565
KEGG	17,450
KOG	25,787
Pfam	34,156
Eggnog	29,498
Swiss-Prot	38,933
Nr	39,411
All	39,521

**Table 4:** Summary of functional annotation for the non-redundant unigenes of *R. simsii.*

Databases	Unigene Number	300~1000 bp		≥1000 bp	
		Number	Percentage	Number	Percentage
NR	39,411	2171	5.51%	37,230	94.47%
GO	18,565	1521	8.19%	17,040	91.79%
COG	16,102	800	4.97%	15,300	95.02%
eggNOG	38,933	2115	5.43%	36,811	94.55%
KEGG	17,450	1109	6.36%	16,336	93.62%
KOG	25,787	1249	4.84%	24,534	95.14%
Pfam	34,156	1699	4.97%	32,454	95.02%
Swiss-Prot	29,498	1521	5.16%	27,971	94.82%
All	39,521	2201	5.57%	37,310	94.41%

**Table 5:** The best 15 pathways annotated by the KEGG database.

No.	Name of Pathway	Pathway ID	No of Transcripts (%)	
1.	Carbon metabolism	ko01200	605	(3.47%)
2.	Protein processing in endoplasmic reticulum	ko04141	608	(3.48%)
3.	Biosynthesis of amino acid	ko01230	525	(3.01%)
4.	Spliceosome	ko03040	700	(4.01%)
5.	Ribosome	ko03010	491	(2.81%)
6.	RNA transport	ko03013	558	(3.20%)
7.	Starch and sucrose metabolism	ko00500	377	(2.16%)
8.	Plant hormone signal transduction	ko04075	470	(2.69%)
9.	Oxidative phosphorylation	ko00190	280	(1.60%)
10.	Glycolysis/gluconeogenesis	ko00010	322	(1.85%)
11.	Plant pathogen interaction	ko04626	324	(1.86%)
12.	mRNA surveillance pathway	ko03015	469	(2.69%)
13.	Ubiquitin mediated proteolysis	ko04120	321	(1.84%)
14.	Amino sugar and nucleotide sugar metabolism	ko00520	271	(1.55%)
15.	Endocytosis	ko04144	251	(1.44%)

## Data Availability

Data are available at the SRA portal (http://www.ncbi.nlm.nih.gov/bioproject/678851 (accessed on 20 March 2021)) of NCBI, accession number: PRJNA678851.

## Supplementary Materials

The following are available online at https://www.mdpi.com/article/10.3390/plants10040649/s1, Table S1: Summary of the predicted alternative splicing (AS) events, Table S2: Summary of lncRNA predicted by CPC, CNCI, CPAT, and Pfam protein structure domain analysis, Table S3: Summary of identified TFs, Table S4: Summary of the transcripts annotated to the reference canonical pathways in the KEGG database, Table S5: Summary of the genes in anthocyanin biosynthesis pathway.
